# Alpha fetoprotein antagonises benzyl isothiocyanate inhibition of the malignant behaviors of hepatocellular carcinoma cells

**DOI:** 10.18632/oncotarget.12407

**Published:** 2016-10-03

**Authors:** Mingyue Zhu, Wei Li, Junli Guo, Yan Lu, Xu Dong, Bo Lin, Yi Chen, Xueer Zhang, Mengsen Li

**Affiliations:** ^1^ Hainan Provincial Key Laboratory of Carcinogenesis and Intervention, Hainan Medical College, Haikou 571199, Hainan Province, P.R. China; ^2^ Key Laboratory of Molecular Biology, Hainan Medical College, Haikou 571199, P.R. China; ^3^ Institution of Tumour, Hainan Medical College, Haikou 570102, Hainan Province, P.R. China; ^4^ Undergraduate Student of Clinical Medicine, Hainan Medical College, Haikou 571199, P.R. China

**Keywords:** alpha-fetoprotein, benzyl isothiocyanate (BITC), hepatocellular carcinoma, apoptosis

## Abstract

Benzyl isothiocyanate (BITC) is a dietary isothiocyanate derived from cruciferous vegetables. Recent studies showed that BITC inhibited the growth of many cancer cells, including hepatocellular carcinoma (HCC) cells. Alpha-fetoprotein (AFP) is a important molecule for promoting progression of HCC, in the present investigation, we explore the influence of AFP on the role of BITC in the malignant behaviours of HCC cells, and the potential underlying mechanisms. We found thatBITC inhibited viability, migration, invasion and induced apoptosis of human liver cancer cell lines, Bel 7402(AFP producer) and HLE(non-AFP producer) cells *in vitro*. The role of BITC involve in promoting actived-caspase-3 and PARP-1 expression, and enhancing caspase-3 activity but decreasing MMP-2/9, survivin and CXCR4 expression. AFP antagonized the effect of BITC. This study suggests that BITC induced significant reductions in the viability of HCC cell lines. BITC may activate caspase-3 signal and inhibit the expression of growth- and metastasis-related proteins; AFP is an pivotal molecule for the HCC chemo-resistance of BITC.

## INTRODUCTION

The preventive effects of cruciferous vegetables, such as broccoli, cabbage, and cauliflower, against cancer have been suggested to be due to their relatively high glucosinolate content, which releases biologically active isothiocyanates (ITCs) [1, 2], ITCs play inhibited roles in the detoxification of carcinogens [3, 4]. Benzyl isothiocyanate (BITC) is a hydrolysis compound of glucotropaeolin in cruciferous vegetables. Many studies have reported that BITC prevents cancer in laboratory animals and may be chemoprotective in humans. Studies have shown that BITC displays anti-tumour activities in many types of tumors, including breast cancer [5, 6], prostate cancer [7], and hepatoma cells [8, 9]. These data imply that BITC can be applied to prevent and treat cancer.

Hepatocellular carcinoma (HCC) is the sixth most common cancer and the third most common cause of cancer death [10, 11]. Although an increasing number of new methods are being applied to treat HCC patients, surgery and chemotherapy are still the most important therapeutic approaches [12]. HCC cells are often refractory to standard chemotherapy and resistant to chemotherapy and radiotherapy [13]. Recurrence or metastasis is quite common in these patients (even in patients undergoing liver cancer resection or chemotherapy), and the survival ratio is only 30% to 40% at 5 years postoperatively [14]. One major mechanism by which many cancer cells develop resistance to chemotherapy is known as multidrug resistance; This phenotype is characterized by diminished intracellular drug accumulation or inactivation of apoptotic signalling, leading to treatment failure [15]. Malignant behaviors, such as drug resistance, migration and invasion, are important characteristics of HCC cells that lead to the poor prognosis of HCC patients. Alpha-fetoprotein (AFP) is early biomarker for the diagnosis of HCC occurrence. Recently, studies revealed that AFP played an important role in promoting proliferation and exhibited anti-apoptotic activity in HCC cells [16–18]. These finding suggest that high expression of AFP was a critical factor that causing the poor prognosis of HCC patients. HCC refractory disease is associated with cancer recurrence and metastasis. In this study, we focused on the effects of AFP on the BITC-induced inhibition of hepatoma cell growth and the migration/invasion potential, explored the effects of BITC on hepatoma cell drug resistance, metastasis and related mechanisms.

## RESULTS

### BITC inhibited HCC cells viability and AFP antagonized the effect of BITC

**Figure 1:**
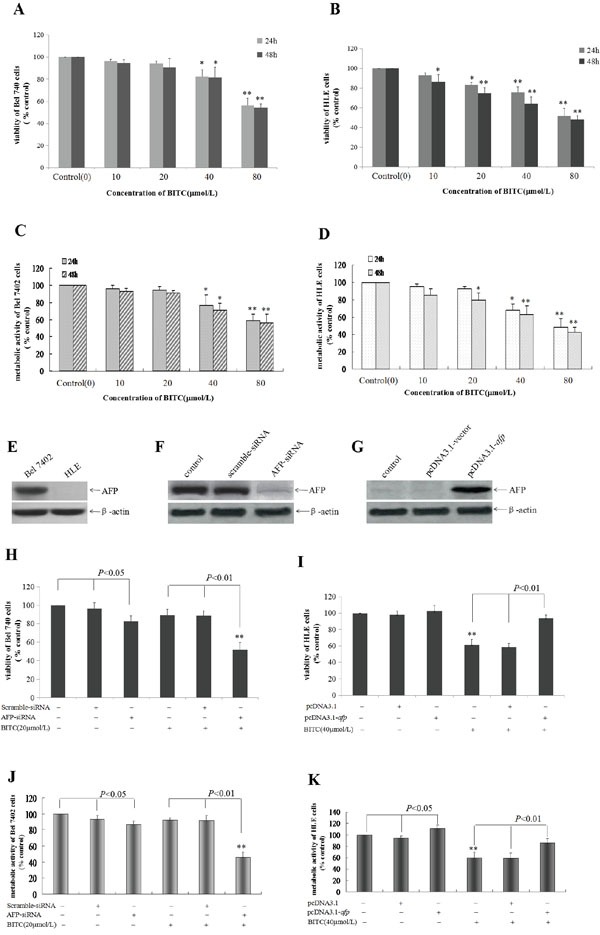
Influence of BITC on Bel 7402 cells and HLE cells viability and the effect of AFP on the role of BITC **A.** and **B.** Bel 7402 cells(A) and HLE cells(B) were treated with different concentrations (10-80 μmol/L) of BITC for 24 hours or 48 hours. Trypan blue exclusion dye assay was used to analyze the cellular viability, *P<0.05 and **P<0.01 vs control group; **C.** and **D.** Bel 7402 cells(C) and HLE cells(D) were treated with different concentrations (10-80 μmol/L) of BITC for 24 hours or 48 hours. The MTT method was applied to detect metabolic activity of the cells. *P<0.05 and **P<0.01 vs control group. **E.** The basal expression of AFP in Bel 7402 cells and HLE cells was detected by Western blotting. **F.** Bel 7402 cells were transfected with the scramble-siRNA vectors and AFP-siRNA vectors for 48 hours, the expression of AFP in Bel 7402 cells was detected by Western blotting. **G.** HLE cells were transfected with the pcDNA-3.1 vectors and pcDNA-*afp* vectors for 48 hours, the expression of AFP in HLE cells was detected by Western blotting. **H.** and **J.** Bel 7402 cells were transfected with the scramble-siRNA vectors and AFP-siRNA vectors for 24 hours followed by treatment with 20 μmol/L BITC for 48 hours. The viability of Bel 7402 cells was analyzed by trypan blue exclusion dye(H), and metabolic activity of Bel 7402 cells was detected by MTT method(J). **P<0.01 vs control groups, scramble-siRNA treated group, AFP-siRNA treated group. **I.** and **K.** HLE cells were transfected with the pcDNA-3.1 vectors and pcDNA-*afp* vectors for 24 hours followed by treatment with 40 μmol/L BITC for 48 hours. The viability of HLE cells was analyzed by trypan blue exclusion dye(I), and metabolic activity of HLE cells was detected by MTT method(J). **P<0.01 vs control group, pcDNA3.1-vector treated group and pcDNA3.1-*afp* treated group. N=6.

In the present study, we applied the trypan blue exclusion dye and MTT method to detect the effects of BITC on the viability and metabolic activity of the human hepatoma cell lines, Bel 7402 and HLE. The results indicated that BITC inhibited viability and metabolic activity of HCC cells in a dose- and time-dependent manner. When the Bel 7402 cells were treated with different BITC concentrations (10-80 μmol/L) for 24 h and 48 h, cellular viability was significantly inhibited when the BITC concentration was >40 μmol/L, the cellular viability ratios were 82.4%-58.6% following treatment for 24 h and 81.7%-54.1% following treatment for 48 h (Figure 1A); and the metabolic activity inhibition ratios were 23.8%-40.9% following treatment for 24 h and 28.8%-46.0% following treatment for 48 h (Figure 1C). Similarly, when the HLE cells were treated with differential BITC concentrations (10-80 μmol/L) for 24 h, cellular viability was significantly inhibited when the concentration was >20 μmol/L, the cellular viability was 83.4%-51.5%; When the HLE cells were treated for 48 h, cellular viability was significantly inhibited at BITC concentrations >10 μmol/L, the cellular viability was 93.1%-48.3%(Figure 1B). HLE cells were treated with differential BITC concentrations (10-80 μmol/L) for 24 h, the metabolic activity was significantly inhibited when the concentration was >40 μmol/L, the metabolic activity inhibition ratios of 22.1%-51.6%; When the HLE cells were treated for 48 h, the metabolic activity was significantly inhibited at BITC concentrations >20 μmol/L, the metabolic activity inhibition ratios of 20.5%-58.0% (Figure 1D). To analyze the antagonistic role of AFP in BITC inhibiting viability and metabolic activity of HCC cell, the RNA interference and AFP-overexpression vector were constructed, and the trypan blue exclusion dye and MTT assay were performed. The results indicated that high basal expression of AFP in Bel 7402 cells, and non expression of AFP in HLE cells(Figure 1E); When Bel 7402 cells were transfected with AFP-siRNA vectors for 48h, the expression of AFP was significantly suppressed(Figure 1F); When HLE cells were transfected with pcDNA3.1-*afp* vectors for 48h, overexpression of AFP in the cells was emerging(Figure 1G). The cellular viability ratio was 51.7% (Figure 1H) and the metabolic activity inhibited ratio was 54.2% in Bel 7402 cells while transfected with the AFP-siRNA vectors for 24 h followed by treatment with BITC(20 μmol/L) for 48 h. In contrast, the cellular viability ratio was 82.9% and the metabolic activity inhibited ratio was 13.5% for the cells while transfected with the AFP-siRNA vector alone (Figure 1J). Conversely, the cellular viability ratio was 102.5%(Figure 1I) and metabolic activity enhanced ratio was 11.3%(Figure 1K) for HLE cells while transfected with the pcDNA3.1-*afp* vectors alone for 48 h, and the cellular viability ratio was 93.2% and metabolic activity enhanced ratio was 23.9% for cells while transfected with the pcDNA3.1-*afp* vectors followed by treatment with 40 μmol/L BITC for 48 h. The cellular viability ratio was 61.2% and the metabolic activity inhibited ratio was 40.4% following treatment with 40 μmol/L BITC alone(Figure 1I and 1K). These results indicated that BITC suppressed the growth of Bel 7402 and HLE cells in a dose- and time-dependent manner and that AFP antagonized the inhibited effects of BITC on proliferation of HCC cells.

### AFP restrained the BITC-induced apoptosome occurrence in HCC cells

**Figure 2:**
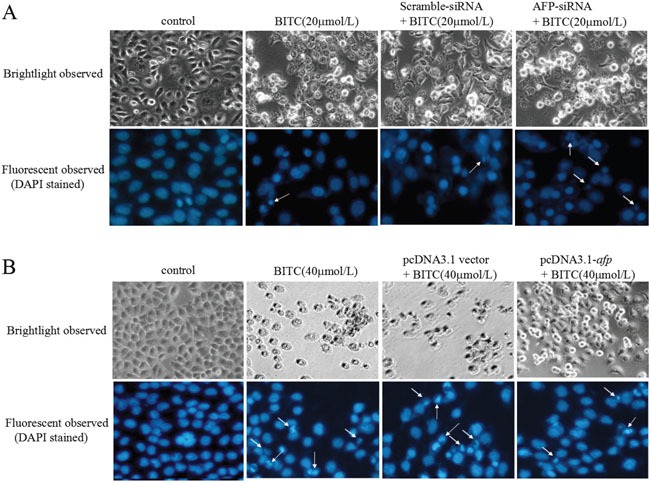
Effects of AFP on BITC regulation of human hepatoma cell growth **A.** Bel 7402 cells were transfected with the scramble-siRNA vectors or AFP-siRNA vectors for 24 hours followed by treatment with 20 μmol/L BITC for 48 hours. Bel 7402 cell growth was observed by microscopy. Bel 7402 cytoblasts were stained with DAPI and observed under a fluorescent microscope. **B.** HLE cells were transfected with the pcDNA3.1 vectors or pcDNA3.1-*afp* vectors for 24 hours followed by treatment with 40 μmol/L BITC for 48 hours. HLE cell growth was observed by microscope. HLE cell cytoblasts were stained with DAPI and observed by fluorescent microscopy. White arrows indicate the apoptosomes. The images were representative of at least three independent experiments.

To investigate whether AFP antagonized the effects of BITC, we performed cell morphological observations. Figure 2A showed that morphological changes occurred in Bel 7402 cells while transfected with the AFP-siRNA vectors for 24 h followed by treatment with BITC(20 μmol/L) for 48 h. The BITC-induced apoptosome occurrence in the Bel 7402 cells was effectively enhanced by silencing AFP expression. Morphological changes were observed in Bel 7402 cells under the fluorescent microscope using DAPI staining. Cellular nuclear condensation and pyknosis were significantly increased and morphological characteristics of apoptosis, including apoptosome formation and nuclear shrinkage, were apparent in the BITC-treated Bel 7402 cells. However, few changes were observed in the cells treated with BITC or the scramble-siRNA alone. Conversely, few apoptotic morphological changes were observed in the HLE cells while transfected with the pcDNA3.1-*afp* vectors followed by treatment with BITC (40 μmol/L). The morphological characteristics of apoptosis, including apoptosome formation and nuclear shrinkage, were significantly decreased in the HLE cells compared to the cells treated with the pcDNA-3.1 vectors or BITC (40 μmol/L) alone (Figure 2B). These results implied that AFP antagonized the BITC-induced apoptosome occurrence in HCC cells.

### AFP inhibited BITC-induced apoptosis of HCC cells

**Figure 3:**
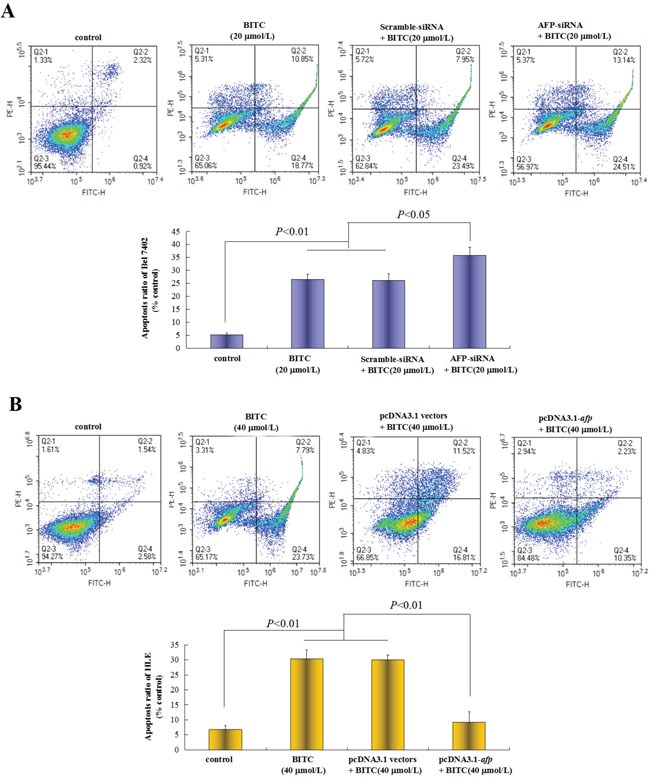
Effects of AFP on BITC regulation of human hepatoma cell apoptosis **A.** Bel 7402 cells were transfected with the scramble-siRNA vectors or AFP-siRNA vectors for 24 hours followed by treatment with 20 μmol/L BITC for 48 hours. Bel 7402 cells apoptosis were analyzed by flow cytometry. The bottom column picture depicts the statistical analysis of the apoptosis ratio. **P<0.01 vs the BITC (20 μmol/L) group and scramble-siRNA group. **B.** HLE cells were transfected with the pcDNA3.1 vectors or pcDNA2.1-*afp* vectors for 24 hours followed by treatment with 40 μmol/L BITC for 48 hours. HLE cells apoptosis were analyzed by flow cytometry. The bottom column picture depicts the statistical analysis of the apoptosis ratio. **P<0.01 vs the BITC (40 μmol/L) group and pcDNA3.1 group. The images were representative of at least three independent experiments.

To evaluate the repressive effects of AFP on BITC-induced HCC cell apoptosis, we applied flow cytometric analysis to detect the apoptosis induced by BITC. Bel 7402 cells were transfected with the AFP-siRNA vectors for 24 h followed by treatment with BITC (20 μmol/L) for 48 h, the apoptosis ratio was (35.7±3.1)%. In contrast, treatment with the AFP-siRNA vectors or BITC (20 μmol/L) alone, the apoptosis ratios were (26.4±2.0)% and (26.0±2.6)%, respectively, these differences were significant (*P*<0.01 for 35.7% vs 26.4% and 26.0%) (Figure 3A). Nevertheless, HLE cells were transfected with the pcDNA3.1-*afp* vectors followed by BITC treatment (40 μmol/L), the apoptosis ratio was 9.1%, whereas treatment with the pcDNA3.1-*afp* vectors or BITC (40 μmol/L) alone, the apoptosis ratios were (30.4±3.0)% and (30.1±1.6)%, respectively, these differences were significant (*P*<0.01 for 9.1% vs 30.4% and 30.1%) (Figure 3B). These results demonstrated that AFP was a critical factor for HCC cells resisting to BITC-induced apoptosis.

### AFP promoted activated-caspase-3 and PARP-1 expression, and suppressed survivin expression and caspase-3 activity in HCC cells

**Figure 4:**
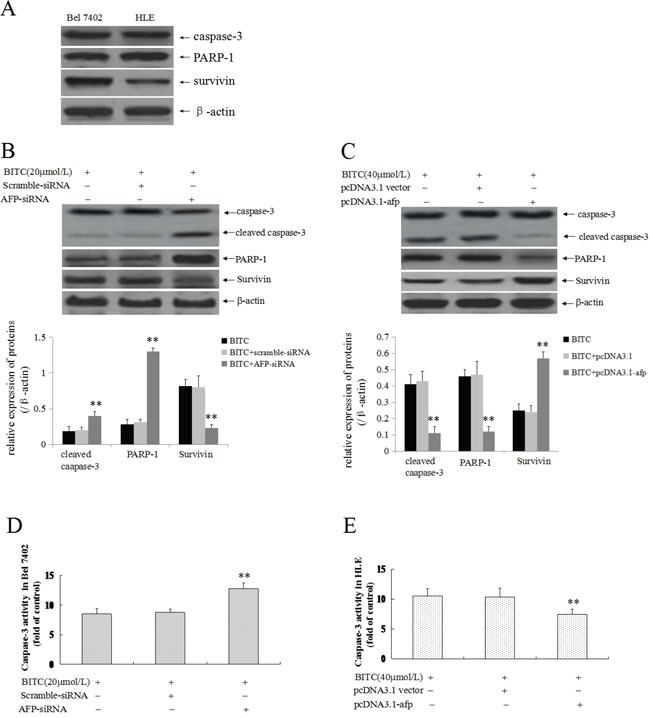
Effects of AFP on BITC regulation of the expression of apoptosis-related proteins and caspase-3 activity in human hepatoma cells **A.** The basal expression of caspase-3, PARP-1 and survivin in Bel 7402 cells and HLE cells were analyzed by Western blotting. **B.** and **D.** Bel 7402 cells were transfected with the scramble-siRNA vectors or AFP-siRNA vectors for 24 hours followed by treatment with 20 μmol/L BITC for 48 hours. Actived caspase-3(cleaved-caspase-3), PARP-1 and survivin expression was evaluated in the cells by Western blotting (B), the bottom column picture depicts the statistical analysis of the relative expression of these proteins. **P<0.01 vs the BITC (20 μmol/L) treated group and BITC (20 μmol/L) plus scramble-siRNA treated group. Activity of Caspase-3 was measured using an enzymatic reaction reagent kit (D). **P<0.01 vs the BITC (20 μmol/L) treated group and scramble-siRNA treated group. **C.** and **E.** HLE cells were transfected with the pcDNA3.1 vectors or pcDNA3.1-*afp* vectors for 24 hours followed by treatment with 40 μmol/L BITC for 48 hours. Actived caspase-3, PARP-1 and survivin expression was evaluated in the cells by Western blotting (C), the bottom column picture depicts the statistical analysis of the relative expression of these proteins. **P<0.01 vs the BITC (40 μmol/L) treated group and BITC (40 μmol/L) plus pcDNA3.1 vectors treated group. Activity of caspase-3 was measured using an enzymatic reaction reagent kit (D). **P<0.01 vs the BITC (40 μmol/L) group and pcDNA3.1 group. The images were representative of at least three independent experiments.

The previous results confirmed that BITC induced Bel 7402 cells and HLE cells apoptosis and that AFP played an antagonistic role. To explore the functional significance of the influence of AFP on the expression of apoptosis-related proteins and caspase-3 activity, we evaluated caspase-3, PARP-1 and survivin expression. In this study, we applied Western blotting method to analyze the basal expression of caspase-3, PARP-1 and survivin in Bel 7402 cells and HLE cells, the results showed that high expression of these proteins in the cells(Figure 4A). Further, Western blotting analysis indicated that activated-caspase-3(cleaved caspase-3) and PARP-1 expression was significantly promoted, whereas survivin expression was significantly restrained (Figure 4B); Caspase-3 activity was significantly stimulated (Figure 4D) in Bel 7402 cells while transfected with the AFP-siRNA vectors followed by treatment with BITC (20 μmol/L) compared to treatment with the scramble-siRNA vectors plus BITC (20 μmol/L) or BITC (20 μmol/L) alone. However, activated-caspase-3(cleaved caspase-3) and PARP-1 expression was significantly suppressed, survivin expression was significantly promoted (Figure 4C), and caspase-3 activity was significantly inhibited (Figure 4E) in HLE cells while transfected with the pcDNA3.1-*afp* vectors followed by BITC treatment (40 μmol/L) compared to treatment with the pcDNA3.1 vectors plus BITC (40 μmol/L) or BITC (40 μmol/L) alone. These results indicated that AFP was able to stimulate survivin expression and suppress activated-caspase-3 and PARP-1 expression, and caspase-3 activity in HCC cells.

### AFP antagonized BITC inhibited migration and invasion of HCC cells

**Figure 5:**
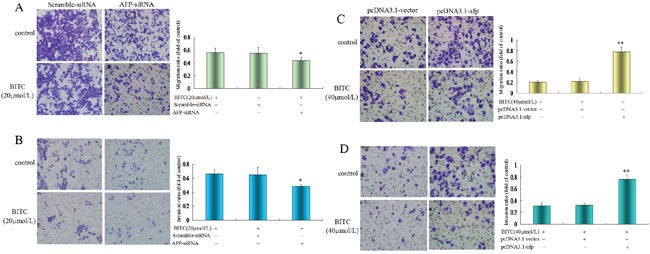
Influence of AFP on BITC regulation of Bel 7402 cells and HLE cells migration and invasion **A.** and **B.** Bel 7402 cells were transfected with the scramble-siRNA vectors or AFP-siRNA vectors for 24 hours followed by treatment with 20 μmol/L BITC for 48 hours. Bel 7402 cell migration (A) and invasion (B) were assessed in the Transwell chamber. *P<0.05 vs the BITC (20 μmol/L) treated group and scramble-siRNA treated group. **C.** and **D.** HLE cells were transfected with the pcDNA3.1 vectors or pcDNA3.1-*afp* vectors for 24 hours followed by treatment with 40 μmol/L BITC for 48 hours. HLE cell migration (C) and invasion (D) were assessed in the Transwell chamber. **P<0.01 vs the BITC (40 μmol/L) treated group and pcDNA3.1 vectors treated group. The images were representative of at least three independent experiments.

A Transwell chamber migration assay was applied to evaluate the influence of AFP on the regulation of Bel 7402 cells and HLE cells migration/invasion by BITC. The migratory and invasive cell ratios were significantly decreased in Bel 7402 cells while transfected with the AFP-siRNA vectors for 24 h followed by treatment with BITC(20 μmol/L) for 48 h. The migratory cell ratio was 0.44±0.04 and the invasive cell ratio was 0.49±0.03 in the AFP-siRNA vectors plus BITC (20 μmol/L)-treated group. In contrast, the migratory cell ratios were 0.56±0.07 and 0.54±0.09, and the invasive cell ratios were 0.66±0.07 and 0.65±0.10 in the BITC (20 μmol/L)-treated group and the scramble-siRNA plus BITC (20 μmol/L)-treated group, respectively; the results were significantly different (*P*<0.05 for 0.44 vs 0.56 and 0.54, Figure 5A, and *P*<0.01 for 0.49 vs 0.66 and 0.65, Figure 5B). However, the migratory cell and invasive cell ratios were significantly increased in HLE cells transfected with the pcDNA3.1-*afp* vectors followed by treatment with BITC(40 μmol/L) for 48 h. The migratory cell and invasive cell ratios were 0.79±0.09 and 0.76±0.07 in the pcDNA3.1-*afp* vectors-treated group, 0.21±0.02 and 0.31±0.05 in the BITC (40 μmol/L)-treated group, and 0.22±0.06 and 0.32±0.03 in the pcDNA3.1 vectors plus BITC (40 μmol/L)-treated group, respectively, these differences were significant (*P*<0.01, 0.77 vs 0.21 and 0.22, Figure 5C, and *P*<0.01, 0.76 vs 0.31 and 0.32, Figure 5D). These results demonstrated that BITC inhibited HCC cell migration/invasion and AFP antagonized the effects of BITC.

### AFP promoted MMP2, MMP9 and CXCR4 expression and activation of MMP2/9 activity in HCC cells

**Figure 6:**
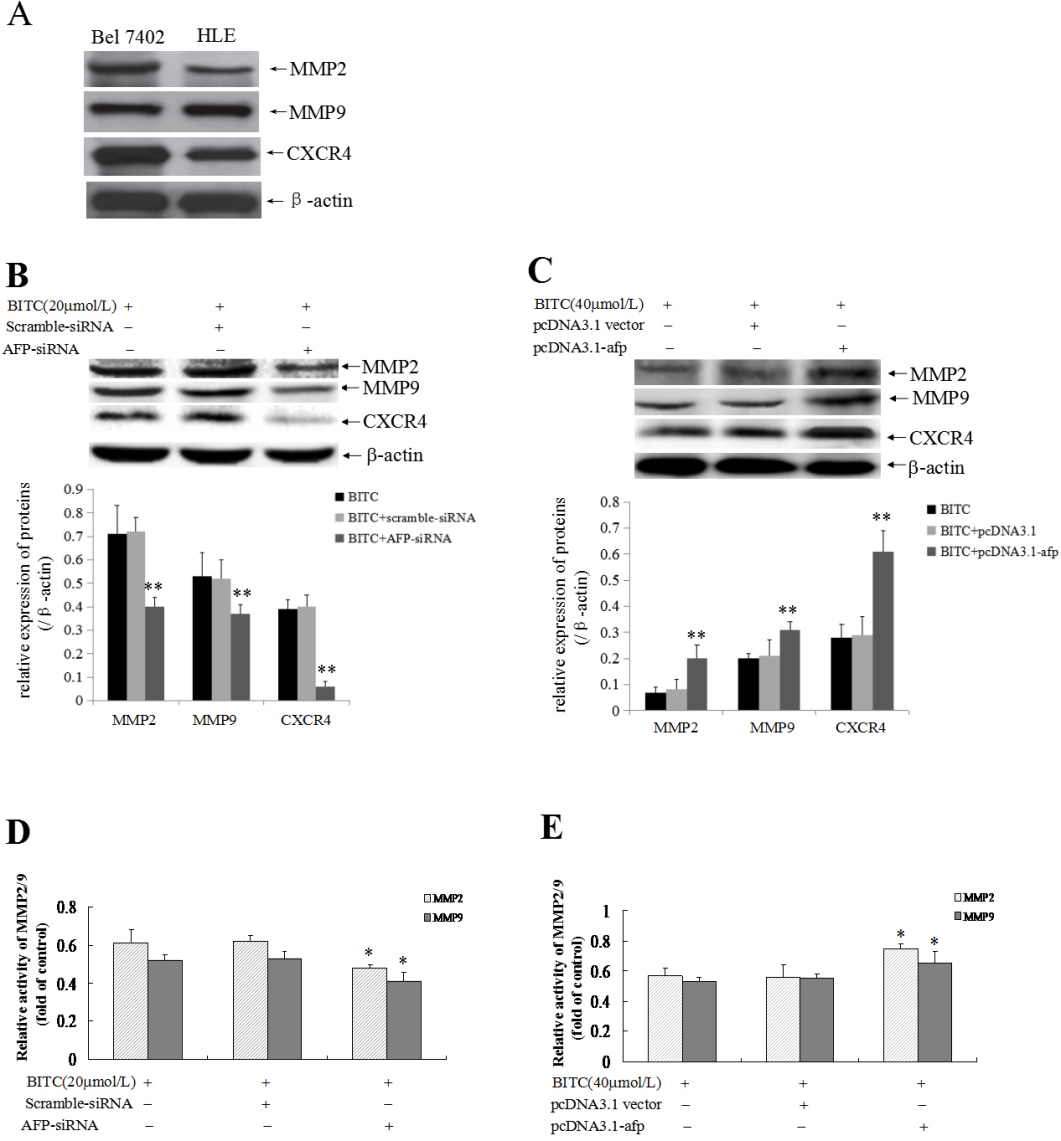
Influence of AFP on BITC regulation of MMP2/9 and CXCR4 expression and MMP2/9 activity in Bel 7402 and HLE cells **A.** The basal expression of MMP2/9, CXCR4 in Bel 7402 cells and HLE cells were analyzed by Western blotting. **B.** and **D.** Bel 7402 cells were transfected with the scramble-siRNA vectors or AFP-siRNA vectors for 24 hours followed by treatment with 20 μmol/L BITC for 48 hours. MMP2/9 and CXCR4 expression in the Bel 7402 cells was detected by Western blotting, the bottom column picture depicts the statistical analysis of the relative expression of these proteins(B). **P<0.01 vs the BITC (20 μmol/L) treated group and BITC (20 μmol/L) plus scramble-siRNA treated group. MMP2/9 activity was analyzed using the gelatine zymography method (D). *P<0.05 vs the BITC (20 μmol/L) treated group and BITC (20 μmol/L) plus scramble-siRNA treated group. **C.** and **E.** HLE cells were transfected with the pcDNA3.1 vectors or pcDNA3.1-*afp* vectors for 24 hours followed by treatment with 40 μmol/L BITC for 48 hours. MMP2/9 and CXCR4 expression in HLE cells was detected by Western blotting, the bottom column picture depicts the statistical analysis of the relative expression of these proteins (C). **P<0.01 vs the BITC (40 μmol/L) treated group and BITC (40 μmol/L) plus pcDNA3.1 vectors treated group. MMP2/9 activity was analyzed using the gelatine zymography method (E). *P<0.05 vs the BITC (40 μmol/L) treated group and BITC (40 μmol/L) plus pcDNA3.1 treated group. The images were representative of at least three independent experiments.

To explore the role of AFP in the expression of metastasis-related proteins and MMP2/9 activity, we evaluated MMP2/9 and CXCR4 expression. Firstly, we applied Western blotting method to detect the basal expression of MMP2/9 and CXCR4 in Bel 7402 cells and HLE cells, the results displayed that high expression of MMP2/9 and CXCR4 in these cells(Figure 6A). Further, Western blotting analysis indicated that MMP2/9 and CXCR4 expression was significantly suppressed (Figure 6B) and MMP2/9 activity was significantly restrained (Figure 6D) in Bel 7402 cells while transfected with the AFP-siRNA vectors for 24 h followed treatment by BITC(20 μmol/L) for 48 h compared to the cells treated with the scramble-siRNA vector plus BITC (20 μmol/L) or BITC (20 μmol/L) alone. Conversely, MMP2/9 and CXCR4 expression was significantly promoted (Figure 6C) and MMP2/9 activity was significantly stimulated (Figure 6E) in HLE cells while transfected with the pcDNA3.1-*afp* vectors for 24 h followed by treatment with BITC(40 μmol/L) for 48 h compared to the cells treated with the pcDNA3.1 vectors plus BITC (40 μmol/L) or BITC (40 μmol/L) alone. These results revealed that AFP harbors a function to stimulate MMP2/9 and CXCR4 expression, and activation of MMP2/9 activity in HCC cells.

## DISCUSSION

HCC cells possess characteristic malignant behaviors, such as drug resistance, metastasis and recurrence, which are important factors for the poor prognosis of HCC patient therapy. Many investigations have suggested that HCC cells are resistant to anti-tumour drug effects [19–21]. Although the drug resistance mechanism of HCC cells involves multiple factors, the pivotal step is unclear. AFP is an early warning biomarker for the diagnosis of HCC occurrence. Recently, studies have shown that AFP plays a critical role in HCC cells resistance to anti-tumour drug effects [22, 23] and AFP expression was positively associated with HCC recurrence and metastasis [24–26]. These results suggested that AFP overexpression was an important factor for the promotion of HCC cells malignant behaviors.

Cruciferous vegetables, such as broccoli, cabbage, and cauliflower, contain unique secondary metabolites called glucosinolates. Glucotropaeolin, which is one of the major glucosinolates of cruciferous vegetables, undergoes myrosinase-dependent hydrolysis to yield BITC. BITC is a promising chemopreventive agent and therapeutic chemodrug for cancer [1, 27]. Previously, many researchers found that BITC not only inhibited proliferation but also suppressed the metastasis and recurrence of cancer cells [28, 29]. BITC also inhibited diethylnitrosamine-induced hepatocarcinogenesis in mice [30]. These results suggested that BITC might possess the ability to suppress the malignant behaviors of cancer cells. Although studies have revealed HCC resistance to anti-tumor drug effects [21, 31], the sensitivity of HCC cells to BITC is undefined. In this study, the trypan blue exclusion dye and MTT results indicated that BITC inhibited the viability and metabolic activity of the human hepatoma cell lines, Bel 7402 and HLE in a dose- and time-dependent manner and exhibited significantly different sensitivity to BITC. The dosage of BITC at 20 μmol/L might suppress viability of HLE cells, but this concentration did not influence the viability of Bel 7402 cells. Silenced AFP expression was able to augment Bel 7402 cell sensitivity to BITC, whereas transfection with an AFP expression vectors could decrease HLE sensitivity to BITC. To explore the effect of AFP on BITC-inhibited apoptosis and metastasis of Bel 7402 cells and HLE cells, we selected 20 μmol/L (this concentration did not inhibit Bel 7402 cell growth) and 40 μmol/L (this concentration significantly inhibited HLE cell growth) for the flow cytometric assay and Transwell chamber analysis. Transfection with the AFP-siRNA vectors followed treatment by 20 μmol/L of BITC could significantly enhance the apoptosis, migration and invasion of Bel 7402 cells, whereas transfection with the pcDNA3.1-*afp* vectors followed by treatment 40 μmol/L of BITC was able to significantly decrease the apoptosis, migration and invasion of HLE cells. These results suggested that BITC inhibited the malignant behaviors of Bel 7402 cells and HLE cells, whereas AFP antagonized these effects of BITC.

Previously, we found that AFP inhibited expression and activity of caspase-3 in HCC cells [32]. AFP also induced expression of Src, Ras, survivin and CXCR4 in HCC cells [33–36]. BITC suppressed apoptosis and cancer cells metastasis by activating caspase-3 activity [37] and restraining CXCR4 [38] and MMP2/9 expression [8]. The results of the present study indicated that silencing AFP expression in Bel 7402 cells might have a synergistic effect with BITC to stimulate activated-caspase-3 and PARP-1 expression, activate caspase-3 activity, and inhibit survivin, MMP2/9 and CXCR4 expression and MMP2/9 activity. AFP overexpression in the HLE cells eliminated the effect of BITC on the inhibition of activated-caspase-3 and PARP-1 expression and caspase-3 activity. Similarly, BITC restrained survivin, CXCR4 and MMP2/9 expression and reversed the effect on MMP2/9 activity. The activation of caspase-3 activity leads to cancer cells apoptosis, and survivin overexpression promotes the growth and drug resistance of cancer cells [39]; Moreover, CXCR4 and MMP2/9 expression may play critical roles in promoting metastasis of HCC cells [40, 41]. This investigation demonstrated that AFP inhibited the expression of apoptosis-related proteins and stimulated expression of metastasis-related proteins, which were the pivotal factors that antagonized the BITC-induced inhibition of the malignant behaviors of HCC cells.

Previous study have found that suppressed expression of AFP maybe inhibit the transduction of PI3K/AKT signal pathway in HCC cells [42], we found that AFP harbored the ability to activate the PI3K/AKT signalling pathway to promote CXCR4, Src, Ras, MMP2/9, and K19 expression and to stimulate the proliferation and metastasis of HCC cells [32, 33–36, 43]. AFP also promoted malignant behaviors and antagonized the apoptosis induced by paclitaxel in hepatoma cells [44, 45]. These findings suggested that AFP was an important molecule for maintaining the malignant behaviors of HCC cells. In this study, we found that BITC inhibited the growth and migration/invasion of Bel 7402 cells and HLE cells. Thus, AFP expression changes in these cells may influence the effects of BITC. To the best of our knowledge, this is the first to report concerning the role of AFP in antagonizing the BITC's cancer suppression effect. Taken together, our observations revealed that AFP suppressed apoptosis through inhibiting the caspase-3 signalling pathway and stimulating the expression of survivin, CXCR4 and MMP2/9 to promote HCC cells viability and migration/invasion. Thus, AFP may be applied as a potential prognostic biomarker and therapeutic target for HCC patients.

### Conclusions

AFP harbors a function to antagonize BITC to suppress HCC cells malignant behaviors. The role mechanism of AFP maybe involve in inhibiting the expression of apoptosis-related proteins and caspase-3 activity, and stimulating the expression of metastasis-related proteins and MMP2/9 activity. Therefore, targeting AFP is a potential strategy for HCC therapeutics.

## MATERIALS AND METHODS

### Cell culture

In this study, we selected the human HCC cell lines Bel 7402 (AFP-producer) and HLE (non-AFP-producer) for the experiments. These cells were the gifts from the Department of Cell Biology, Peking University Health Science Center (Beijing, China), and the cells were cultured with DMEM medium (GIBCO, Carlsbad, CA, USA) supplemented with 10% heat-inactivated foetal calf serum (FCS)(GIBCO, Carlsbad, CA, USA). The cells were incubated at 37°C in a humidified atmosphere containing 5% CO2 as described in a previous study [46].

### Trypan blue exclusion dye and MTT methods to analyze cells viability and metabolic activity

To determine cell viability, Bel 7402 cells or HLE cells were seeded at a density of 2.5×10^4^ cells per well in 6-well plates. Following treated with different BITC concentrations (10-80 μmol/L) for 24 h or 48 h, cell viability was determined by trypan blue exclusion dye assay using Typan Blue Staining Cell Viability Assay Kit (Beyotime Biotech Corp, Haimen, Jiangshu, China). Cells restricting trypan blue entry were considered viable, cellular viability ratio=(control group viable cells-treated groups viable cells)/control group viable cells 100%. To detect cell metabolic activity, Bel 7402 cells or HLE cells were plated at a density of 1.5×10^4^ cells per well in 96-well plates and cultured in RPMI 1640 medium supplemented with 10% FCS at 37°C in a humidified atmosphere with 5% CO_2_ for 48 h. Then, the supernatants of the cultured cells were replaced with medium without FCS for another 24 h. The cells were treated with different BITC concentrations (10-80 μmol/L) for 24 h or 48 h. The effects of BITC (Sigma, USA) on cell growth were measured using the methylthiazolyldiphenyl-tetrazolium bromide(MTT) assay as previously described [47] following a standard procedure. The metabolic activity ratio=(control group *A*_490_-treated group *A*_490_)/ control group *A*_490_ ×100%.

### Cell morphology observations and nuclear DAPI staining

To observe alterations in cellular morphology induced by the BITC treatment, the Bel 7402 cells or HLE cells were plated at a density of 2.0×10^4^ cells/ml in 24-well plates. The cells were treated with 20 μmol/L (Bel 7402 cells) or 40 μmol/L BITC (HLE cells). After treatment for 48 h, the cellular morphology was observed under a microscope. The cells were stained with a 4, 6-diamidino-2-phenylindole dihydrochloride (DAPI) solution and imaged using a fluorescence microscope at a 100× magnification. In this study, nuclear pyknosis and fragmentation were used to define apoptosis; these criteria were evaluated by microscopy as described in previous studies [48, 49]

### RNA interference assay, AFP expression vector construction and transient transfection

The AFP-siRNA vectors and AFP expression vectors (pcDNA3.1-*afp*) were constructed as described in a previous study [49]. Bel 7402 and HLE cells were transfected with the AFP-siRNA vector or the AFP expression vector, respectively, for 24 h and then treated with BITC (20 μmol/L or 40 μmol/L, respectively) for 48 h. The sensitivity of the Bel 7402 cells to BITC and the antagonistic effect of AFP on the viability and metabolic activity of the cells were verified using the trypan blue exclusion dye assay and MTT assay. Apoptosome formation was observed by fluorescent microscopy. Cell apoptosis was evaluated by flow cytometric analysis, and the expression of apoptosis-related proteins and metastasis-related proteins was analyzed using a Western blotting assay.

### Flow cytometry method to analyse HCC cell apoptosis

Bel 7402 cells and HLE cells were cultured in DMEM medium supplemented with 10% FCS at 37°C in a humidified atmosphere with 5% CO_2_. After transfection with the AFP-siRNA vectors or AFP expression vectors, respectively, for 24 h, the cells were treated with BITC (20 μmol/L or 40 μmol/L, respectively) for 48 h. The extent of apoptosis of the Bel 7402 cells and HLE cells was analyzed by flow cytometry. The detailed procedure was described in a previous study [49].

### Cell migration and invasion assay

The cellular migration and invasion assays were performed according to the manufacturer's protocols. To measure cell migration, Transwell chambers were used to observe cultured cell inserts (Transwell chamber; 8-mm pore size; Costar, High Wycombe, UK). Bel 7402 cells and HLE cells were transfected with the AFP-siRNA vectors and pcDNA3.1-*afp* vectors, respectively, for 24 h; Then, the cells were placed into the wells of 12-well culture plates and the upper and lower chambers were separated. The cells (5×10^4^) were added to the upper chamber, cultured with serum-free DMEM medium and treated with BITC (20 μmol/L for the Bel 7402 cells or 40 μmol/L for the HLE cells); the lower chamber was filled with complete medium containing 20% FCS. After 48 h of incubation, the cells in the upper chamber were carefully removed with a cotton swab. The cells that had migrated through the membrane to the lower surface were fixed with 90% methanol and stained with 0.1% crystal violet. The number of cells that had migrated through the pores was quantified by counting five independent visual fields under the microscope (Olympus) using a 20× objective. For the invasion assays, the Transwell chambers were covered with Matrigel (BD Falcon, USA). The experimental procedure was similar to that described for the migration assays. The results were reported as the number of migratory cells or the invasive cell ratio=(numbers of non-treated groups-numbers of treated groups)/numbers of non-treated groups.

### Analysis of caspase-3 activity and gelatine zymography to assess MMP2/9 enzyme activity

Bel 7402 cells were transfected with the AFP-siRNA vectors for 48 h and HLE cells were transfected with the pcDNA3.1-*afp* vectors for 48 h. Then, the cells were treated with BITC (20 μmol/L or 40 μmol/L, respectively). Caspase-3 activity was measured with a commercial kit according to the manufacturer's protocols (APOPCYTO Caspase-3 Colorimetric Assay Kit; Medical and Biological Laboratories, Japan) as described in a previous study [32]. The MMP-2/9 protease activities in the concentrated supernatants from Bel 7402 and HLE cells were detected by zymography. Briefly, SDS-PAGE was performed under non-reducing conditions using gels containing 1% gelatine (Mini-PROTEAN II system; Bio-Rad). The electrophoresis was performed at 4°C. After washing with 2% Triton X-100 to remove the SDS, the gels were incubated in 37°C with buffer containing 50 mM Tris (pH 7.5), 5 mmol CaCl_2_ and 1 μmol/L ZnCl_2_ for 18 h. The MMP2/9 activities were visualized by staining with Coomassie Blue R-250 (Bio-Rad) [50].

### Western blotting analysis

To estimate the influence of AFP on the expression of apoptosis-related proteins and metastasis-related proteins, Bel 7402 cells and HLE cells were transfected with the AFP-siRNA vectors or AFP expression vectors, respectively, for 24 h, followed by BITC treatment (20 μmol/L or 40 μmol/L, respectively) for 48 h. The expression of apoptosis-related proteins, such as activeted-caspase-3, PARP-1 and survivin, and metastasis-related proteins, such as MMP2, MMP9 and CXCR4, in the Bel 7402 cells and HLE cells was analyzed by Western blotting. Briefly, these protein were probed for the following primary antibodies: mouse anti-caspase-3(1:500), PARP1(1:500), survivin(1:500) or β-actin (1:1000); rabbit ant-MMP2(1:400), MMP9 (1:400) or -CXCR4(1:400) antibody (all from Santa Cruz Biotechnology Inc.). The detailed procedure was described in a previous study [19].

### Statistical analysis

Data are presented as the mean ± S.D. The statistical analysis was performed using Student's *t* test (for two experimental groups). Significance was set at *P*<0.05. Statistical significance was determined using Student's t test and the *F* test (SPSS 11.5 software for Windows, SPSS Inc., Chicago, IL, USA).

## Abbreviations

AFP: alpha-fetoprotein
BITC: benzyl isothiocyanate
FCS: foetal calf serum
MTT: 3-(4, 5-dimethylthiazol-2-yl)-2, 5-diphenyltetrazolium bromide
PARP-1: poly(ADP-ribose) polymerase
MMP2/9: matrix metalloproteinase
CXCR4: CXC motif chemokine receptor 4
